# Global development of children’s palliative care: An international survey of in-nation expert perceptions in 2017

**DOI:** 10.12688/wellcomeopenres.15815.3

**Published:** 2020-10-14

**Authors:** David Clelland, Danny van Steijn, Mary Ellen Macdonald, Stephen Connor, Carlos Centeno, David Clark

**Affiliations:** 1 School of Interdisciplinary Studies, University of Glasgow, Dumfries, DG1 4ZL, UK; 2 ATLANTES Global Observatory of Palliative Care, University of Navarra, Pamplona, Navarra, 31008, Spain; 3 Faculty of Dentistry, McGill University and Pediatric Palliative Care Research, Montreal Children’s Hospital, McGill University Health Centre, Montreal, Quebec, Canada; 4 Worldwide Hospice Palliative Care Alliance, London, WC1X 9JG, UK

**Keywords:** palliative care, children's palliative care, global development, mapping

## Abstract

**Background: **The growing interest in tracking the global development of palliative care provision is not matched by research on the development of palliative care services specifically for children. Yet it is estimated that worldwide, 21 million children annually could benefit from the provision of palliative care. We report on a global study of children’s palliative care development and offer suggestions for further improvement in design and method.

**Methods: **Primary data on the level of children’s palliative care development in 2017 was collected from in-country experts through a specific question in an online questionnaire that sought to measure the overall level of palliative care provision globally. Countries were assigned to one of six categories on the basis of the responses obtained. Conflicting responses from the same country were resolved with reference to a hierarchy of preferred respondents.

**Results: **Our data allowed the categorisation of 113 countries, accounting for 65% of the global population aged under 20. Number of countries (% of global child population) in each category were as follows: 1) no known activity, 21 (4%); 2) capacity-building, 16 (24%); 3a) isolated provision, 55 (30%); 3b) generalized provision, 5 (1%); 4a) preliminary integration into mainstream provision, 14 (8%); 4b) advanced integration, 7 (2%).

**Conclusions: **Children’s palliative care at the highest level of provision is available in just 21 countries, accounting for fewer than 10% of the global population aged under 20. It is concentrated in high income settings, whilst the majority of the global need for such care is in low- and middle-income countries. Our study is a useful tool for global advocacy relating to children’s palliative care and a stimulus for the creation of improved indicators to measure it at the country level.

## Introduction and context

### Children’s palliative care – global context

The World Health Organization (WHO) has defined palliative care for children as ‘the active total care of the child's body, mind and spirit, and also involves giving support to the family’ ^1^. The WHO states that this should begin when a life limiting condition is diagnosed and should continue whether or not the child receives treatment directed at the disease. It requires health providers to evaluate and alleviate the child’s physical, psychological, and social distress; and to be effective, it should be supported by a broad interdisciplinary approach that includes the family and makes use of available community resources. WHO maintains that palliative care for children can be successfully implemented even if resources are limited and can be provided in tertiary care facilities, in community health centres, as well as in children’s own homes ^2^.

One-third of the global population is aged under 20 years. This is the age range to which WHO applies the idea of children’s palliative care in the context of life-limiting and life-threatening conditions. Yet whilst 98% of the global need for children’s palliative care occurs in low- and middle-income countries, the majority of such care is available only in high-income countries ^3^. It is estimated that around the world, 21 million children annually could benefit from some form of palliative care ^4^. The number includes more than 8 million children with complex problems that require specialised attention. Of these the most common difficulties relate to perinatal conditions, followed by congenital anomalies, Human Immunodeficiency Virus (HIV)/Acquired Immune Deficiency Syndrome (AIDS), and cancer ^5^.

Currently we know very little about the scale of development of children’s palliative care services in the global context. This stands in contrast to the growing body of information and research that is plotting patterns of growth and development around the world in palliative care for adults. We therefore focus here on this knowledge deficit relating to children’s palliative care, report on a recent study that has shed some light on the issues, and reflect on the methodological and conceptual problems involved in research of this type. While the evidence we present is far from definitive, we see it as an important contribution to a crucial and emerging field of enquiry.

### Mapping global palliative care development

For more than a decade, a series of studies has monitored the level of palliative care development in all the countries of the world - beginning in 2006 ^6^, followed up in 2011 ^7^ and most recently updated for 2017 ^8^. These studies have contributed significantly to advocacy, planning and monitoring for the improvement of palliative care worldwide. They have also sought progressively to develop more robust methods for measuring the country-level status of palliative care provision, through continuing identification and refinement of the appropriate indicators ^9^. The first study allocated each country to one of four categories of development, using data synthesised from a variety of academic, professional and ‘grey’ literature, with expert opinion used as a substitute where necessary. The second study used a refined six category classification and was based on the identification of in-country experts or ‘champions’ who were asked for their opinion on their country’s level of development. The most recent iteration of the study develops the method further, again using six categories of development, but now with more detailed information gathered from a survey of national experts across 198 countries, and based on a set of 10 palliative care indicators derived from the literature.

### Children’s palliative care – current evidence

Despite these advances in measuring the overall level of palliative care provision globally, there remains limited evidence on the development of children’s palliative care. The first two global mapping studies, for example, attempted to measure only the overall level of palliative care development in each country, with no specific focus on services for children.

Knapp *et al.* ^10^, however, looking specifically at palliative care for children, used a systematic review of peer-reviewed and other published literature, as well as information on numbers of what the authors term ‘paediatric specific organizations’, to assign countries to the four categories of overall palliative care development used in the first global mapping study. This placed only 11 countries at Level 4, the highest category of palliative care development, while 19 countries were identified as being at Level 3 (isolated provision) and 36 at Level 2 (capacity building with no operational services). The majority of countries – 126 (65.6%) – were categorised as being at Level 1, indicating that there was no reported evidence of children’s palliative care. Although this process used two reviewers to assign levels of provision, with further review by an expert panel, the work nonetheless shared the limitations of the 2006 global study in relying on published data (limited to English-language literature) supplemented by expert opinion, sometimes from outside the country.

### Aim of this work

The aim of the work presented here was to obtain a first global overview of the national development of children’s palliative care based on the knowledge of in-country experts. With this in mind, we included two specific questions in the third global mapping study that focussed, respectively, on the level of development of palliative care for children and on its implications.

The paper proceeds as follows. First, we set out the research methods and design of our most recent study, along with the protocol for assigning each country, where possible, to one of six categories of children’s palliative care development. Second, we present the results of this process and compare them to the relevant countries’ overall level of palliative care provision and to the results of the study of Knapp *et al.* Third, we discuss the strengths and limitations of our approach, with suggestions for future improvement.

## Methods

### Ethical statement

The study was approved by the University of Glasgow College of Social Sciences Research Ethics Committee. Approval was granted on 15 January 2018 (Application No: 400170065). Prior to data collection, the purpose of the study was explained to all participants and written consent for participation in the study was obtained from each participant before access to the questionnaire was granted. Participants were informed that they had the right to withdraw from the study at any point in time, without any repercussions.

### Data collection

The design and methods of the third global palliative care mapping study have been presented in detail ^11^. Data were collected via an online questionnaire completed by in-country experts actively engaged at the national level in the development, delivery and co-ordination of palliative care activity. These respondents were identified in consultation with international and regional palliative care associations. The survey was administered in 198 territories, comprising the 193 Member States of the United Nations (UN), two Observer States, along with Kosovo, Somaliland, and Taiwan, China. A total of 560 experts from 179 (90%) countries for which contacts could be found were asked to complete the questionnaire. For 19 (10%) countries we were unable to identify an expert contact.

### Categorisation

While the questionnaire included multiple questions linked to a variety of indicators that enabled us to assess the overall level of national palliative care development, two questions focused specifically on the level of development of palliative care provision for children. Respondents were asked “Which of the following categories best describes palliative care activity related to children in your country?” A set of detailed category descriptors was then provided ( Table 1).

**Table 1.** Six levels of children’s palliative care development.

**Category 1:** No known palliative care activity for children	A country in this category is one where current research reveals no evidence of any palliative care activity relevant specifically to children.
**Category 2:** Capacity building palliative care activity for children	A country in this category shows evidence of wide-ranging initiatives designed to create the organisational, workforce, and policy capacity for the development of palliative care services for children, although no service or specific program has yet been established. There are some developmental activities including attendance at, or organisation of, key conferences, personnel undertaking external training in palliative care, lobbying of policy makers and Ministries of Health, and emerging plans for service development.
**Category 3a:** Isolated children’s palliative care provision	A country in this category is characterized by the development of children’s palliative care activism that is still patchy in scope and not well-supported; sources of funding that are often heavily donor-dependent; limited availability of morphine; at least a service or program can be identified by other professionals in the country as a best practice model for palliative care for children; there are a few children’s palliative care services or specific programs, but they are limited in relation to the need of the population.
**Category 3b:** Generalised children’s palliative care provision	A country in this category is characterized by the development of children’s palliative care activism in several locations with the growth of local support in those areas; multiple sources of funding; the availability of morphine; several hospice-palliative care services or programs for children from a range of providers; and the provision of some training and education initiatives by the hospice and palliative care organizations.
**Category 4a:** Children’s palliative care services at a preliminary stage of integration into mainstream health care services	A country in this category is characterized by the development of a critical mass of children’s palliative care activism in a number of locations; a variety of palliative care providers and types of services and programs; awareness of palliative care on the part of health professionals and local communities; the availability of strong pain relieving drugs other than morphine; some impact of palliative care on policy; the provision of a substantial number of training and education initiatives by a range of organizations; and the existence of a national palliative care association.
**Category 4b:** children’s palliative care services at an advanced stage of integration into mainstream health care services	A country in this category is characterized by the development of a critical mass of children’s palliative care activism in a wide range of locations; comprehensive provision of all types of children's palliative care by multiple service providers; broad awareness of children’s palliative care on the part of health professionals, local communities, and society in general; unrestricted availability of morphine and most strong pain-relieving drugs; substantial impact of children’s palliative care on policy; the existence of children’s palliative care guidelines; the existence of recognized education centres and academic links with universities with evidence of integration of children’s palliative care into relevant curricula; and the recognition of children’s palliative care by a national association that has achieved significant impact.

A follow up question – “Please provide here any additional information on children’s palliative care services in your country you would like us to know” – allowed respondents to add more information or comments about the level of children’s palliative care.

**Figure 1.**
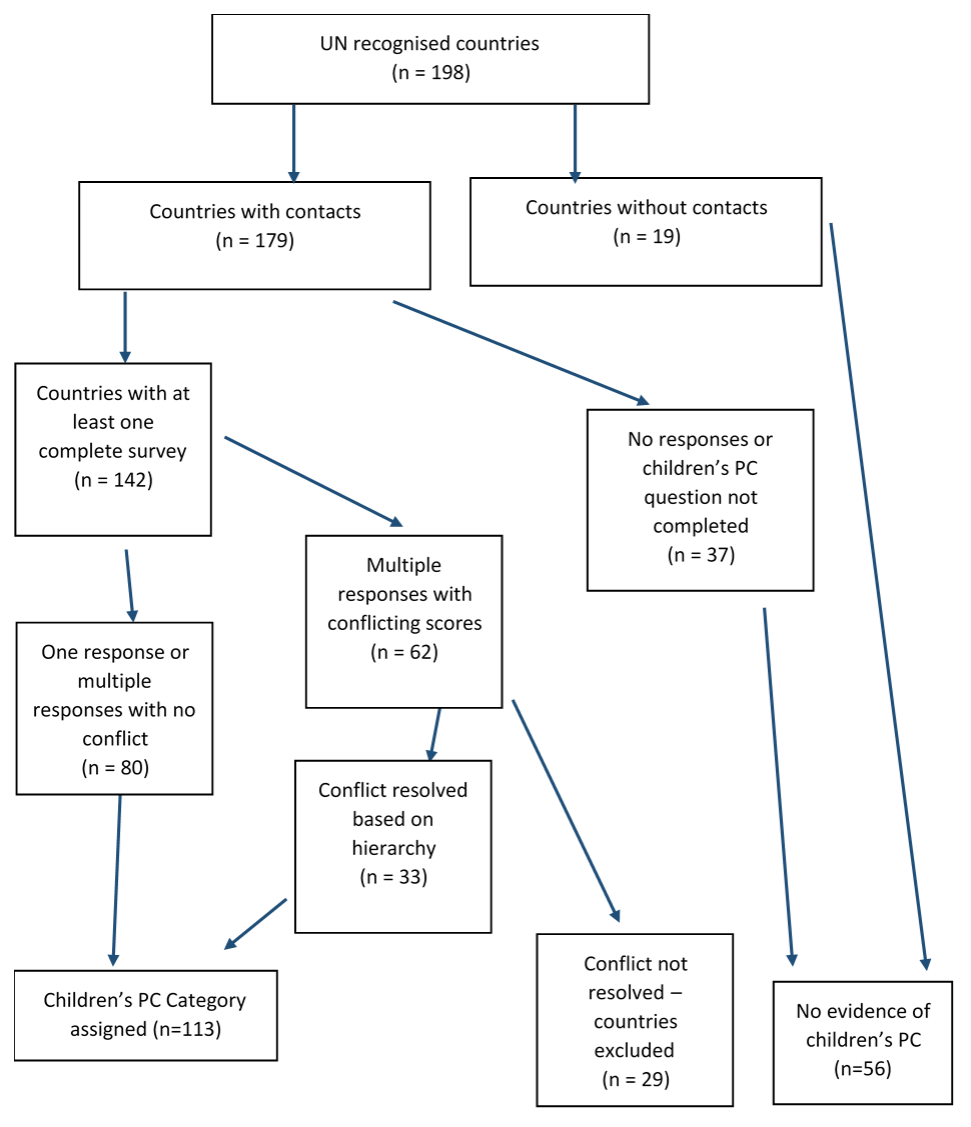
Categorisation process. PC, palliative care.

Figure 1 explains the categorisation process. Where there was only one completed response per territory, or where there were multiple responses that were in agreement, the territory was allocated a category of children’s palliative care development on that basis. Where there were two or more responses with conflicting ** assessments of the most appropriate category, these were resolved where possible by privileging the assessments of those survey participants closest to the top of the following hierarchy:

I) Representatives of the national in-country hospice-palliative care association or nearest professional association (e.g. society for palliative medicine or hospice forum). The person should have an established administrative and/or leadership role in the organisation, making them a reliable source of information.
II) Academic experts with known interests and research experience in hospice-palliative care development in-country and/or beyond, as evidenced by peer-reviewed publications. The person should have an established academic role in hospice-palliative care research or education, making them a reliable source of information.
III) Policy specialists (in or outside government) with experience of and/or responsibility for hospice-palliative care delivery in-country. The person should have an established policy role relating to hospice-palliative care, making them a reliable source of information.
IV) Palliative care representatives, academics or policy specialists from outside the country but with direct knowledge of its hospice-palliative care provision, making them a reliable source of information.

Where it was not possible to resolve conflicts on this basis, that is where survey participants offering differing assessments of their country’s level of children’s palliative care had similar roles – then the country was excluded from the analysis.

## Results

### The global development of children’s palliative care

**Figure 2.**
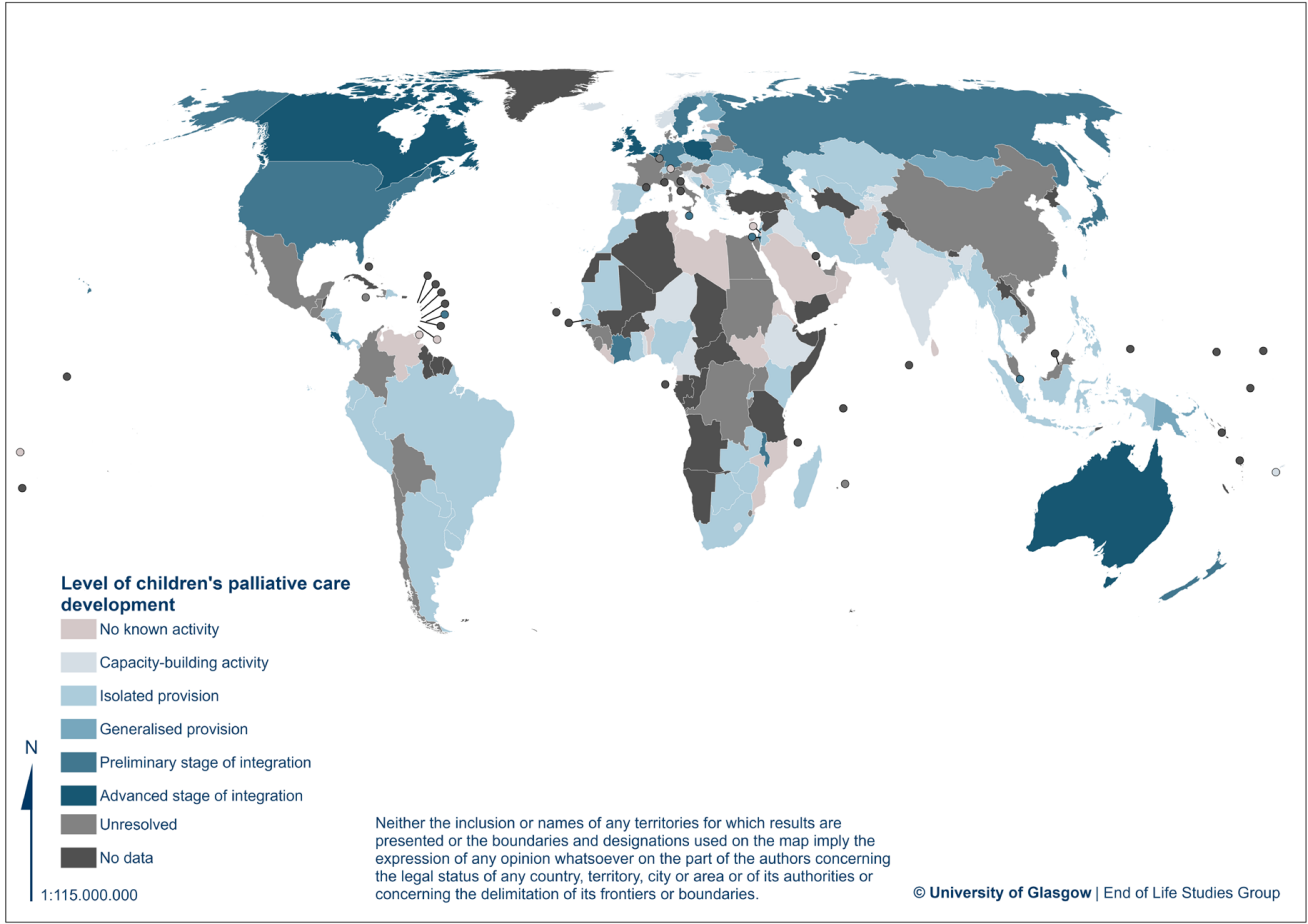
Level of children´s palliative care development.

Using this process, an assessment of the level of children’s palliative care development was reached for 113 (57%) of the 198 countries included in the study ( Table 2, Figure 2). These 113 countries account for 65% of the total global population of children – defined here as being aged 0–19 in line with the WHO convention, so including infants, children and adolescents.

**Table 2.** Countries by level of children’s palliative care development.

Category	Countries (n=198) (%)	Children population (millions of children aged under 20) (% of global)	Countries
(1) No known palliative care activity for children	21 (10.6%)	92M (3.6%)	Afghanistan, Benin, Cyprus, Equatorial Guinea, Eritrea, Estonia, Grenada, Liberia, Libya, Liechtenstein, Mozambique, Oman, Palestine, Samoa, Saudi, Arabia, Serbia, South Sudan, Sri Lanka, Trinidad & Tobago, Tunisia, Venezuela
(2) Capacity building palliative care activity for children	16 (8.1%)	610M (24.1%)	Cameroon, Croatia, Ethiopia, Fiji, Iceland, India, Iraq, Kyrgyzstan, Lesotho, Lithuania, Niger, Norway, Portugal, Slovenia, Tajikistan, Togo
(3a) Isolated children’s palliative care provision	50 (25.3%)	763M (30.1%)	Albania, Argentina, Azerbaijan, Bangladesh, Bosnia & Herzegovina, Botswana, Brazil, Bulgaria, Cambodia, Czech Republic, Dominican Republic, Ecuador, Georgia, Ghana, Greece, Honduras, Indonesia, Iran, Jordan, Kazakhstan, Kenya, Kuwait, Lebanon, Macedonia, Madagascar, Mauritania, Moldova, Morocco, Myanmar, Nepal, Nicaragua, Nigeria, Pakistan, Panama, Paraguay, Peru, Philippines, Romania, Rwanda, Senegal, Slovakia, South Africa, South Korea, Spain, Switzerland, Thailand, Uruguay, Uzbekistan, Zambia, Zimbabwe
(3b) Generalisedchildren’s palliative care provision	5 (2.5%)	16M (0.6%)	Finland, Latvia, Mongolia, Papua New Guinea, Ukraine
(4a)children’s palliative care services at preliminary stage of integration into mainstream health care services	14 (7.1%)	190M (7.5%)	Barbados, Côte d’Ivoire, Germany, Israel, Japan, Malawi, Malta, Netherlands, New Zealand, Russia, Singapore, Sweden, Taiwan(China), USA
(4b)children’s palliative care services at advanced stage of integration into mainstream health care services	7 (3.5%)	42M (1.7%)	Australia, Belgium, Canada, Costa Rica, Ireland, Poland, United Kingdom
Conflicting data from survey returns (unresolved) (See Supplementary Table 1, *Extended data* ^13^)	29 (14.6%)	646M (25.5%)	Armenia, Austria, Belarus, Bolivia, Burundi, Chile, China, Colombia, Congo (DR), Denmark, Egypt, El Salvador, France, Guatemala, Guinea, Haiti, Hungary, Italy, Jamaica, Luxembourg, Malaysia, Mauritius, Mexico, Sierra Leone, Sudan, Swaziland, Uganda, United Arab Emirates, Vietnam
No data from survey returns	56 (28.3%)	173M (6.8%)	Algeria, Andorra, Angola, Antigua & Barbuda, Bahamas, Bahrain, Belize, Bhutan, Brunei, Burkina Faso, Cape Verde, Central African Republic, Chad, Comoros, Congo (Republic), Cuba, Djibouti, Dominica, Gabon, Gambia, Guinea-Bissau, Guyana, Kiribati, Kosovo, Laos, Maldives, Mali, Marshall Islands, Micronesia, Monaco, Montenegro, Namibia, Nauru, North Korea, Palau, Qatar, Saint Lucia, San Marino, Sao Tome e Principe, Seychelles, Solomon Islands, Somalia, Somaliland, St Kitts & Nevis, St Vincent & the Grenadines, Suriname, Syria, Tanzania, Timor l’Este, Tonga, Turkey, Turkmenistan, Tuvalu, Vanuatu, Vatican City, Yemen

Note: Population for number of people aged 19 and under (2017) by country from UN Population Database. Available at https://population.un.org/wpp/Download/Archive/Standard/

**Figure 3.**
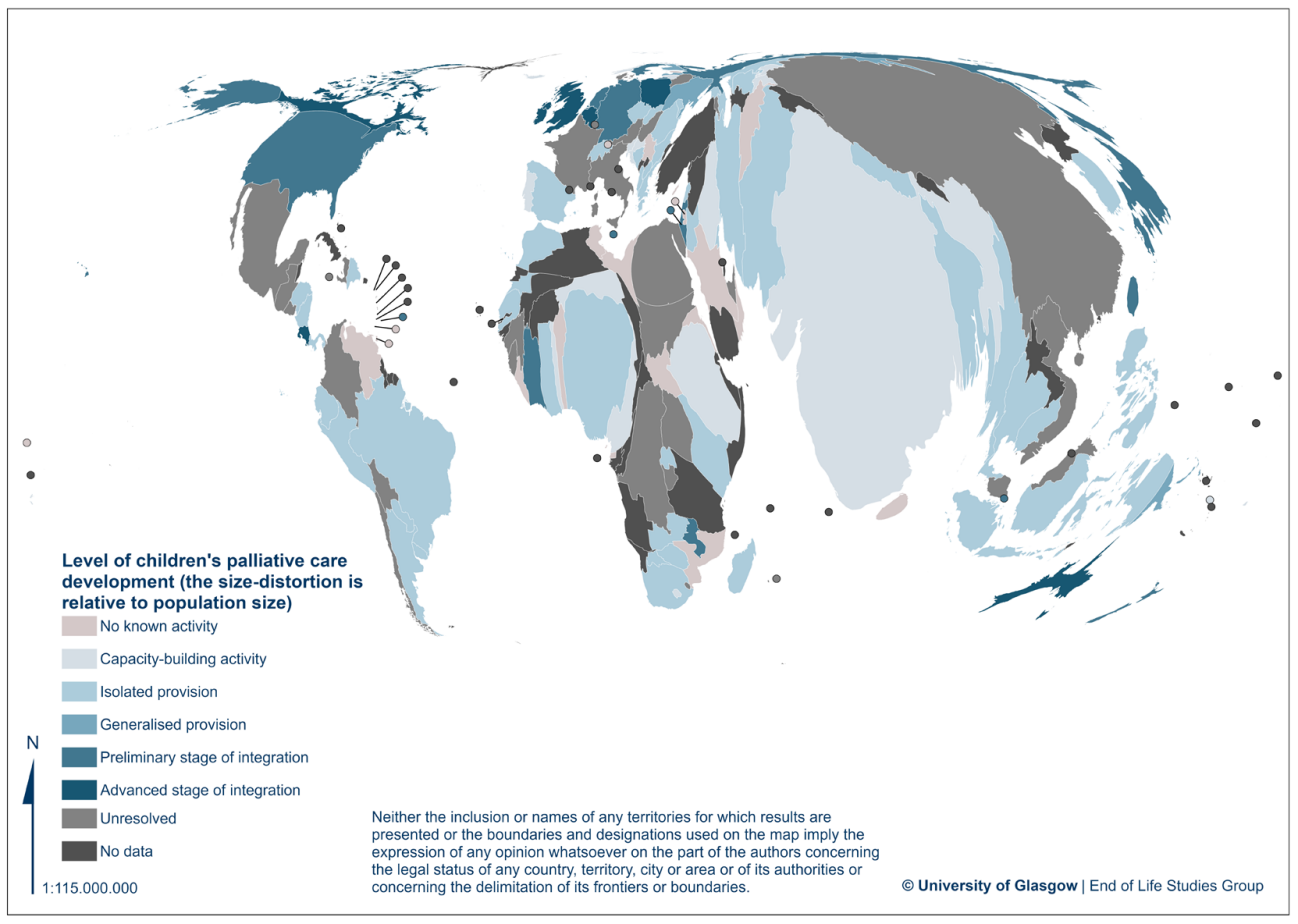
Level of children´s palliative care development with a cartogram of the population size.

In Figure 2 and Figure 3 we present world maps showing the distribution of the different levels of children’s palliative care development. The cartography was made using ArcGIS Pro (ESRI), version 2.6. The layer used for representation is ‘Admin 0 – Countries, 1:50m’, obtained from Natural Earth. For both maps the equal earth projection was used, which maintains the relative size of areas across the map. For Figure 2 a blue-scale choropleth map was used to visualize the level of development of children’s palliative care and a reddish colour was used to denote ‘no known activity’. For Figure 3 the same choropleth map was used in combination with a cartogram made by the Gastner-Newman algorithm ^12^. The cartogram resizes the countries relative to their population, resulting in countries with high population being bigger in size. This is used to emphasise the overrepresentation of population in countries with palliative care levels of ‘isolated provision’ or lower. Dots were used in both maps to visualize various small countries that were included in the study.

Only seven countries, accounting for 42M children (1.7% of the world total), were placed in Category 4b, with the most advanced level of integrated children’s palliative care. A further 14 countries were in Category 4a (190M, 7.5%), with preliminary integration of children’s palliative care into mainstream services.

Five countries were in Category 3b, indicating the presence of generalised provision of children’s palliative care (16M, 0.6%), and 50 in Category 3a (763M, 30.1%) with isolated provision.

In Category 2 were 16 countries, with evidence of some capacity building activity in children’s palliative care (610M, 24%).

In the lowest category (1) were 21 countries with no reported children’s palliative care activity. In addition, we added to this category 28 countries for which no survey was returned, and 28 countries for which the children’s palliative care question was not completed, on the basis that this suggests there is no evidence of any palliative care specifically for children in these countries. Taken together, the countries in this expanded category 1 account for 265M children aged under 20, 11% of all the world’s children.

For a further 29 countries, there were multiple responses that provided a conflicting assessment of the level of children’s palliative care, and where this conflict could not be resolved with reference to the hierarchy set out above. They were therefore not assigned a category. These account for 646M children, 26% of all the world’s children. In the majority of cases these had survey responses from two or three different in-country experts (in two cases there were more than three responses). In 14 of the 29 countries, assessments of the level of children’s palliative care varied by one category, and in 10 cases by two categories. The remaining five countries had more significant conflicts, with respondents disagreeing by three or four categories (See Supplementary Table 1, *Extended data* ^13^).

### Comparison with overall palliative care level

There is a broad alignment between countries’ overall categorisation of palliative care development, already reported ^7^ and based on a range of indicators, and their specific categorisation for children’s palliative care as presented here. Table 3 shows that all of the countries in Category 4b for children’s palliative care are also in 4b overall, as are 10 of the 14 countries in Category 4a for children’s palliative care. There is, however, no firm relationship between the national level of overall palliative care development and that of children’s services. There are a number of outliers, in particular a small number of countries in category 4b overall that are in categories 1 or 2 for the level of children’s palliative care. In addition, the overwhelming majority of those countries for which there were no data on children’s palliative care were placed in the lowest category overall, indicating no known or reported palliative care activity.

**Table 3.** Number of countries by children’s palliative care and overall palliative care category.

	Children’s PC Level									
**Overall PC Level**		**4b**	**4a**	**3b**	**3a**	**2**	**1**	**No** **data**	**Unres-** **olved**	**Total**
	**4b**	7	10	1	3	4	1	0	4	30
	**4a**	0	3	2	10	0	0	0	6	21
	**3b**	0	1	1	8	1	4	3	4	22
	**3a**	0	0	1	28	9	10	5	12	65
	**2**	0	0	0	1	0	4	5	3	13
	**1**	0	0	0	0	2	2	43	0	47
	**Total**	7	14	5	50	16	21	56	29	198

**PC, palliative care.**

Only two of the countries with responses that reported no children’s palliative care activity were also placed in the lowest category for their level of overall palliative care development. On the other hand, of those countries for which no information on children’s palliative care was returned, the vast majority (43/56) were in the lowest category overall.

### Comparison with other country level indicators

This data can also demonstrate the relationship between the level of children’s palliative care development and the United Nations Human Development Index (UNHDI), World Bank Income Level (WBIL), Universal Health Coverage Index (UHCI) and WHO region. Of the seven countries at the highest level of children’s palliative care development, all are at high or very high levels of UHCI, WBIL, and in the top two quintiles of the UHCI. Four are in Europe, two in the Americas and one in the Western Pacific Region (See Supplementary Table 2, *Extended data* ^13^).

### Comparison over time

Since we did not collect specific information on children’s palliative care in the earlier world mapping studies of palliative care development, we have no basis for comparison over time with our current findings. However, some indication is possible using the country-level classifications assigned by Knapp *et al.*, for 2011, which were based on the categories used in the first mapping study. Table 4 shows how the 113 countries for which a positive categorisation was possible on the basis of our 2017 survey, were classified (on a four-category scale) by Knapp *et al.* Most obviously, the majority of those countries in Categories 1, 2 and 3a in 2017 were placed in Category 1 by the Knapp *et al.* study. This reflects the high proportion of countries – over half – for which Knapp *et al.* found no evidence of children’s palliative care provision. Given the differences in methodologies, it is not possible to say with certainty the degree to which the results from our present study represent genuine changes in children’s palliative care development over the intervening period.

**Table 4.** Comparison of 2017 and 2011 country categorisations of children’s palliative care development.

	2011 Children’s PC Level (Knapp *et al.*)						
		1	2	3	4	NC	Total
**2017 Children’s PC** **Level (Clark *et al.*)**	**1**	17	1	1	0	2	21
	**2**	10	5		1	0	16
	**3a**	29	11	9	1	0	50
	**3b**	2	1	2	0	0	5
	**4a**	3	5	1	4	1	14
	**4b**	1	0	2	4	0	7
	**Total**	62	23	15	10	3	113

**PC, palliative care; NC, no categorisation.**

***Free text commentary.*** Respondents were given the opportunity to add more information about the development of children’s palliative care in their country in an open text box. Table 5 shows some indicative quotes from respondents, arranged by their country’s categorisation, that demonstrate the type of commentary provided. Respondents provided broad overviews of institutional frameworks and social contexts, further detail on the number and type of medical facilities and specialists providing palliative care for children, the geographical spread of these services, and narratives about the progress of children’s palliative care development in their country.

**Table 5.** Respondents’ comments on children’s palliative care.

Category	Comments
**1**	We dispense some measure of palliative care in some of our hospitals. We admit palliative care cases, manage and discharge, although there are no specialists, no morphine, no registry, no home visits etc. It is not comprehensive.
	Children can benefit from the Palliative Care Units existing in [neighbouring country]
	There are not any activities for palliative care for children in my country.
	Palliative care for children is usually provided by the programmes providing palliative care for adults. No specific paediatric palliative care programmes are currently available in the country.
	There is only onechildren’s general hospital. This hospital provides non-specialized palliative care services to children in need e.g. HIV/AIDS and malnutrition.
**2**	Activities are begun by NGOs in some places. Professionals (doctors, nurses, management) in the oncological department in [capital city] Children’s Hospital make efforts to establish children’s palliative care. There is a symposium about children’s palliative care held every year. Also, the recognition of children’s palliative care by the national association has achieved a significant impact. There is also an active palliative care committee at the Ministry of Health.
	We created this year a hospital committee for children’s palliative care; decrees have been signed for the national hospital and we are now working on different protocols to formalize them.
**3a**	There is only one children’s onco-hematological hospital. This offers chemotherapy and symptom control for children in need, but there is not any palliative care or hospice for children in the country.
	There are a few complete palliative care services but they are in big cities. Palliative care for children is growing fast but in a very heterogeneous way at the national level. There are provinces/states that do not have any paediatricians trained in palliative care and other provinces who have several teams in big cities.
	There are several specialized paediatric onco-hematological hospitals that provide specialized palliative care services for children.
	There is only one NGO providing home palliative care to children and young adults as well as psychological support to parents, siblings and relatives.
	There is only one paediatric palliative care service; it is a well-established service; provides 24/7 service with inpatient and outpatient plus one home team The service is only for the patients that are seen at the centre, mostly cancer patients. Trying to help neighbouring countries by providing training to physicians and nurses.
	Progressing slowly. Health care professionals need to be sensitised and trained to refer and support children with palliative care needs. Still lagging behind adult service provision particularly in rural areas.
	The children’s palliative care services are currently limited to two University Teaching Hospitals but there is a Government willingness and plan to spread the services to District Hospitals in the nearest future.
	Hospitalized children suffering for cancer have a palliative care support program but this is generally missing for other specialties.
**3b**	The Ministry of Social Affairs and Health recommendations for organizing palliative care includes a recommendation for building up children palliative care network and services. The official group for implementation of these recommendations and another group to revise the legislation/need for a new legislation of end-of-life and palliative care include plans for children’s palliative care. University Hospitals have home care teams for children, but in general there is no official plan for children palliative care so far.
	There are palliative care standards at national level in children’s care. Palliative care specialists are trained, before they are certified, both in children’s and adult palliative care on a one year course.
**4a**	Services for children are provided by the hospital, and upon discharge, quality care is given. It should be noted that there is not a high percentage of children in need of palliative care. Should a case arise, families are very keen to give primary care with our monitoring support.
	All 6 departments of paediatric oncology in the leading university hospitals have developed an advanced service for palliative care.
	We have one children’s hospice. Some children are taken care of in advanced home care teams around the country.
	In most cases families take the total responsibility and provide the end of life care to their dying child. Despite availability of opiates in country, there isn’t proper system of prescribing and consuming opiates for reducing pain of patients. Parents get pain killers, even sometimes opiates, on an ‘over the counter’ basis from pharmacies to reduce pain of their loved one.
**4b**	One children’s hospice that serves the country; children’s palliative care team in the national children’s hospital; consultant- provided service in one other large maternity hospital; care in other hospitals provided by 'champion paediatricians' (i.e. paediatricians with an interest in palliative care) with the support (either direct or indirect) from the palliative care team in the national children’s' hospital' children in the community cared for in every local health office area by adult community palliative care teams and local primary care and paediatric teams with support of the 10 Clinical Nurse Coordinators for Children with Life-Limiting Conditions’. Four consultants (2.2 whole time equivalents) provide children’s palliative care services – two consultants are paediatricians with specialist palliative care training; two consultants are adult physicians with a special interest in children’s palliative care. children’s palliative care recognised in national model of care for children.
	Seven free-standing residential hospices but many programs in all paediatric health centres

Comments have been edited for clarity and confidentiality NGO, non-governmental organisation.

## Strengths and limitations

The research presented here is the most comprehensive attempt yet to map the country-level development of children’s palliative care around the world. In basing this assessment on responses from in-country palliative care experts from 113 territories, the method moves beyond a reliance on published data and the judgement of external parties. As part of a broader study of global palliative care, this therefore represents a significant contribution to evidence and understanding about the spread of palliative care provision for children.

Nevertheless, there remain a number of limitations with this approach and these should be addressed in future research.

- Although this method – based on the assessment of ‘in-country’ experts with detailed knowledge of palliative care in their territory – can be considered more robust than previous approaches, categorisation on this basis is nevertheless informed to some extent by the professional judgements of survey participants. Such self-reported judgements cannot be independently verified, and some participants may have over- or under-estimated their country’s levels of development. There are, for example, instances of countries at the highest level of overall palliative care development, of human development and income that have been identified as having a very low level of children’s palliative care development. The deficiencies of this approach could therefore potentially be strengthened through triangulation with other sources of evidence, though we lacked the resources to do this in the present study. However, while confidentiality principles do not allow us to give more details, many survey responses came from impeccable organisational sources and were signed off by senior persons, sometimes of global standing in the field of palliative care, and very well placed to provide an assessment of the situation in their country.
- Furthermore, some readers may raise the question of relevant expertise in the respondents to our survey, and take the view that national experts in palliative care will not necessarily have in-depth knowledge of palliative care services available for children. This is a reasonable assertion but could be subject to challenge, particularly in poorer countries where there is a less developed divide between ‘adult’ and ‘children’s’ palliative care. We acknowledge the distinct and perhaps contested status of children's palliative care in our discussion of definitional issues below; given this, we propose that a survey specifically about children's palliative care would be the desirable next step in developing understanding of this area. It would, however, be unreasonable to discard the assessments of these palliative care experts on the grounds that they might not be specialists in children's palliative care, especially when the latter may not even be present in some countries.
- Related to this, the survey question did not define ‘palliative care activity related to children in your country’, meaning there was the potential for the question to be interpreted in different ways, influenced by different understandings of what constitutes palliative care and who are considered to be ‘children’ in different national contexts. We explore this in more detail in the section below on matters of definition.
- A significant proportion of countries included in the global mapping survey have been excluded from these results on the basis that participants provided conflicting categorisations that could not be reconciled. The exclusion of these countries limits the extent to which the results presented here can be considered a comprehensive global assessment of palliative care development. While there are no straightforward ways of addressing this, a number of possible approaches may be considered. At its simplest, this could take the average value of multiple categorisations. Alternatively, more detailed information could be sought about the characteristics of survey participants to allow a judgement to be made on which were best placed to assess their country’s level of development, or the views of external experts could be sought to moderate the proposed categorisations. We do not consider this approach self-defeating, but regard it as an honest and open statement of limitation which we mitigated by not including the relevant countries in the final analysis.
- In some instances, it was not possible to identify palliative care experts in-country, and in others, where eligible respondents were identified, they did not complete the questionnaire or the specific question relating to children’s palliative care.
- Data limitations were compounded by language constraints. Questionnaires were only available in three European languages (English, Spanish and French). This both impeded communication with reluctant respondents and might also have added to the barriers for non-English speakers willing to complete the questionnaire. Terminological issues may also have been further exacerbated by translation factors.

Some readers may also question the grading assigned to their own country in our analysis. In previous iterations of the world map of palliative care, where expert opinion has been sought, we have been gratified by how few of such concerns have been raised. In the present study, and within the bounds of research ethics approval, we would be happy to have dialogue with those questioning their country grading, or indeed who may be able to shed light on countries where we did not give a grade due to conflicting returns from in-country respondents.

We have given careful thought to how a future study might be made more robust than our current attempt. One starting point for this would be to establish a specific collaboration with experts in children’s palliative care development, around the world, building on our existing links with the Worldwide Hospice Palliative Care Alliance ^14^. Such a collaboration might be with an organisation like the International Children’s Palliative Care Network (ICPN) ^15^. This approach has already yielded good results in the WHO-Europe context ^16^. Individuals in the collaboration would then constitute a panel for a bespoke survey of children’s palliative care in each country. In some countries it will be difficult to identify such experts, but this would be a finding in itself. The indicators to be used in such a survey would be determined by use of a consensus process with the collaborative members. Thereafter and in parallel with the in-nation expert survey, it would also be possible to survey, in partnership with WHO, representatives from national governments who hold responsibility for children’s palliative care. The ‘experts’ survey might concentrate more on indicators of service delivery and outcomes, education and the ‘culture’ of children’s palliative care. The ‘government’ survey might give greater weight to policies, service availability, outcomes and workforce capacity. Such approaches might still require moderation of the data, and would require more investigative techniques, with feedback to and further questioning of respondents with regard to ‘conflicting’ data. We are optimistic about this line of development, which has been prompted by reviews of earlier versions of our paper.

## Matters of definition

Children’s palliative care is a complex domain that is similar to, but also distinct from, adult palliative care. Understanding the specificities of children’s palliative care, and situating it globally, requires attention to the gamut of life-threatening and life-limiting illnesses that can affect children and youth, myriad culturally diverse conceptions of childhood, and the vast range of national, ethnic, and legal contexts of care provision.

The diagnostic categories, illness trajectories and prognosis that fall under the umbrella of children’s palliative care are vast, with significant variation in severity, symptoms, prognosis, and disease trajectories. Some illnesses requiring children’s palliative care are ‘life limiting’ (i.e., those with no reasonable hope of cure and from which children will die); others are ‘life threatening’ (i.e., those for which curative treatment may be feasible but may fail). These conditions have been divided into the following four categories for the purposes of research and to better focus care: 1. Life-threatening conditions (e.g., infectious diseases, cancer, organ failure and transplant, long-term ventilation); 2. Conditions in which premature death is inevitable but intensive treatment can prolong life (e.g., cystic fibrosis, Duchenne muscular dystrophy); 3. Progressive conditions without curative treatment options (e.g., metabolic conditions, incurable cancer); 4. Irreversible non-progressive conditions causing severe disability (e.g., cerebral palsy, brain or spinal cord injury) ^17^.

Children’s palliative care therefore includes a greater diversity of illnesses and a longer duration of survival than adult palliative care ^18^. Many of these children live with physical, neurological and cognitive impairments, therefore requiring intensive family and/or institutionalized caregiving ^17^. Advances in rehabilitation and disability studies have shifted focus beyond functional impairments to better appreciate the interplay between a child’s capacities and the accommodations available in their local environment.

Conventional biomedical ‘paediatric’ models parse the category of childhood into neonates, infants, children, and adolescents based upon psychological developmental models. Children’s palliative care extends beyond these categories starting with the unborn child (e.g., perinatal palliative care) and into young adulthood (e.g., many children will live with life-limiting illnesses beyond 19 years old). Further, local cultural conceptions of children and youth need to be considered when developing children’s palliative care services. Advances in childhood studies have challenged stage-based conceptions of child development for their simplistic depictions of children’s understandings of death and dying, favouring instead conceptions of children’s experiences centred on the recognition of children as active agents with morally meaningful concerns about their lives and the people around them ^19, 20^.

Children’s palliative care, as defined by leading authorities – e.g., the WHO ^21^, and the UK’s Together for Short Lives ^17^ – is attentive to the family as the unit of care, offering support for the child as embedded within a family unit. This support starts with diagnosis, and proceeds throughout the illness, to end-of-life care and death, and beyond into the bereavement period. Understanding who is important to the child’s life and care (e.g., extended family, parents, sibling, community), and at what age and for which kinds of treatments the child should be afforded some agency, will depend on local logics, legal systems, and cultural practices.

While conventions in the global north are to provide children’s palliative care through inter-professional and interdisciplinary teams, services in rural contexts as well as in low- and middle-income countries are often without access to specialist care. Instead, care is often provided through child-focused general health professionals, with the assistance of adult-specific palliative care specialists if available ^22^.

All of these dimensions challenge our ability to map and categorise levels of children’s palliative care development at the national level and globally. Significant further work is needed to generate indicators of children’s palliative care development that result in measurable outcomes but at the same time incorporate the nuanced and complex world of palliative care for children. The next step in developing a more comprehensive and robust map of global children’s palliative care provision would therefore be a bespoke global survey online, adapting the approach taken in the global studies of palliative development and making use of new and more imaginative indicators, which capture the complexities of defining palliative care for children, the varied age groups and development categories to which it applies, and the specific forms of co-production that may be involved in its design and delivery.

## Conclusions

We have been able to identify a total of just 21 countries in the highest categories of development for children’s palliative care (7 in 4b and 14 in 4a); these countries contain 232 million children and young people age 19 and under (9.2% of the global total).

In addition, 778 million children (30.7%) live in 55 countries with only isolated and patchy provision

Meanwhile, 610 million children (24.1%) live in 77 countries that only have capacity building activity in place.

A further 265 million children (10.4%) live in 106 countries where no known children’s palliative care activity is taking place, or it has proved impossible to gather any evidence on it. An even greater number, 646 million children (25.5%), live in 29 countries where the available evidence on the level of children’s palliative care development is contradictory.

Accordingly, less than 10% of those under 20 years old live in countries where palliative care for children is of the highest current standards. Almost a third of children live in countries where such care is highly limited in provision, and incommensurate with need. One quarter of all children live in countries that are only beginning to mobilise efforts for children’s palliative care. The rest live in countries where it is proving difficult even to assess available levels of children’s palliative care.

We have previously shown that the overall development of palliative care, despite some gains over time, is incapable of meeting existing need. For the world’s children who could benefit from palliative care, the situation is even more serious and requires a global intervention of massive scale for its rectification.

We contend that the in assessing the limitations of our study, the perfect cannot be the enemy of the good. We know, based on our experience of previous mapping studies we have conducted, that despite the limitations and our gradual efforts to overcome them, these studies have proved enormously valuable to the field. Indeed, they constituted the underlying evidence base for the World Health Assembly Resolution on palliative care, which was passed in 2014, and which of course supports the provision of palliative care across the entire life course. No other group has attempted to map the global development of children’s palliative care in the manner we present in our paper. Until such an attempt is made with an improved methodology, ideally by an international team of specialists in the field of children’s palliaitve care, we argue that the current work should be seen, not as definitive, but as the best evidence we currently have, and which moreover is highly likely to be of benefit to the field on which it is focussed. To take a phrase from the paediatrician Winnicott, it is ‘good enough’. 

## Data availability

### Underlying data

Enlighten: Research Data: Global development of children’s palliative care: the picture in 2017: http://dx.doi.org/10.5525/gla.researchdata.995 ^13^

This project contains the following underlying data:

- SurveyResponsesQ26_995.xlsx
- NB in response to one review of Version 1 who commented on one element in the underlying data as an example of a problem with this data, we noted a survey response wrongly assigned to the USA on the basis that it came from the representative of an organisation that works in one of the poorest countries in the world, but who for some reason gave the USA as the host country for the organisation – an issue we noted in some other responses. We dealt with these case by case in the analysis, but have amended the relevant record in the underlying data.

Data are available under the terms of the Creative Commons Attribution 4.0 International license (CC-BY 4.0).

The authors are unable to make all data publicly available because of the terms of data sharing included in the consent process for this study. Furthermore, it is not possible to effectively de-identify all the participants and their organisations in the free-text responses to the survey. However, access will be granted on a case-by-case basis upon request to the corresponding author ( david.clelland@glasgow.ac.uk). Access to the full underlying data will be granted upon request from a researcher for the purposes of further research providing the requesting researcher is in possession of a protocol that has been approved by an ethics committee and which satisfies the guarantees of anonymity originally given to the research participants.

### Extended data

Enlighten: Research Data: Global development of children’s palliative care: the picture in 2017: http://dx.doi.org/10.5525/gla.researchdata.995 ^13^

This project contains the following extended data:

- Supplementary Table 1: Conflicting Assessments of Children’s Palliative Care Development (DOCX)
- Supplementary Table 2: Level of Children’s Palliative Care Development by Country-Level Indicators (DOCX)

Data are available under the terms of the Creative Commons Attribution 4.0 International license (CC-BY 4.0).

## Disclaimer

Neither the inclusion or names of any territories for which results are presented or the boundaries and designations used on the map imply the expression of any opinion whatsoever on the part of the authors concerning the legal status of any country, territory, city or area or of its authorities or concerning the delimitation of its frontiers or boundaries.
