# Supplementary material for: Global development of children’s palliative care: An international survey of in-nation expert perceptions in 2017
Source: Wellcome Open Res. 2020 Oct 14;5:99. Originally published 2020 May 20. [Version 3] doi: 10.12688/wellcomeopenres.15815.3 (PMC8042514; doi:10.12688/wellcomeopenres.15815.3)
Supplement: Peer review history

# Peer review: Reviewer response for version 3

**DOI:** 10.21956/wellcomeopenres.17970.r43258

**Published:** 2021-04-12

**Authors:** Michael J McNeil, Justin Baker

**Affiliations:** 1 St. Jude Children's Research Hospital, Memphis, TN, USA; 2 Division of Quality and Life and Palliative Care, Department of Oncology, St. Jude Children's Research Hospital, Memphis, Tennessee, USA

In this manuscript “Global development of children’s palliative care: An international survey of in-nation expert perception in 2017,” the authors aim to assess the status of children’s palliative care around the world. Overall, I think this is an incredibly valuable and important contribution to the literature in understanding the progress in advancing pediatric palliative care around the world and the profound deficit that still exists between high income countries and low- and middle-income countries where the majority of children live. As mentioned, this is a well-written manuscript that would benefit from addressing the following issues:

**Introduction:**

- This is a well-written and strong introduction which introduces the need of pediatric palliative care (PPC) in resource-constrained settings and the current inequity in how PPC is distributed around the world. Also, the authors lay out the fact that while advancements are being made globally in palliative care, there is a paucity of evidence in the arena of pediatric specific palliative care. I will note however, that there are a few more references to consider for the introduction with regards to PPC in the global context. These references will supplement and enhance the introduction by describing in greater depth the current status of PPC globally. I would particularly highlight the Global Atlas of Palliative Care at the End of Life from the WPCA. You reference the WPCA later in the manuscript and in the Global Atlas from 2014 figure 38 and supplemental table II they describe countries in various levels similar to this work.

1. Connor, S.R. *et al*. Global Atlas of palliative care at the end of life. ^1^
2. Caruso Brown, A.E. *et al*. Reported availability and gaps of paediatric palliative care in low- and middle-income countries: a systematic review of published data. ^2^
3. Downing, J. *et al*. Children’s palliative care in low- and middle-income countries. ^3^

**Methods:**

- This was an incredibly ambitious effort and I applaud the authors for this work. With regards to the in-country experts, what qualified an individual as an “in-country expert”? They are described as individuals engaged in the development, delivery and co-ordination of palliative care at the national level and the hierarchy was well described. However, in reading through this I did not see clinicians who are actively practicing in the country of interest who may have particular insights in the actual care delivery on the ground, especially when it comes to care delivery for children.
- The second comment is with regards to the response rate. We have the response rate from a country level (113 of 179 countries) but we do not have a response rate of the 560 experts. This is an important piece of information when thinking about survey-based research methodologies.

**Results: **

- No comments as they are well presented and described.

**Strengths and Limitations**

- The authors have done well in addressing many of the limitations associated with this particular study and highlight the fact the deficit of not having pediatric specific experts as an area of further focus. Additionally, within the matters of definition it is critical to highlight the differences in cultures and views of who would be considered “children.”
- Additionally, I would add that as mentioned in the WHO definition, quality pediatric palliative care includes support of the family and what is defined as the family unit is also unique between cultures and individual units.

**Conclusions:**

- There is great discussion about the need for further research in understanding the gap of pediatric palliative care globally and ways to ameliorate those research gaps. However, there is less discussion about the actual gap in clinical care for children with life-threatening conditions. While further research is needed, this paper describes the deficit, and it would be helpful ways to scale up palliative care for children in resource-limited settings.
- Finally, there is a grammatical error in the first sentence of the last paragraph “We contend that the in assessing the limitations of our study, the perfect cannot be the enemy of the good.”

In summary this is an extremely important topic that I believe is a valuable contribution to the literature. While there have been significant advancements in palliative care globally and pediatric palliative care in high-income settings there is a significant deficit in LMICs. The authors do a solid job of presenting their data without overstating the findings and identify ways of focusing of pediatric palliative care expertise with collaborations with WHPCA and ICPCN among others.

Is the work clearly and accurately presented and does it cite the current literature?

Yes

If applicable, is the statistical analysis and its interpretation appropriate?

Yes

Are all the source data underlying the results available to ensure full reproducibility?

Yes

Is the study design appropriate and is the work technically sound?

Yes

Are the conclusions drawn adequately supported by the results?

Yes

Are sufficient details of methods and analysis provided to allow replication by others?

Yes

Reviewer Expertise:

Pediatric Palliative Care, Pediatric Oncology.

We confirm that we have read this submission and believe that we have an appropriate level of expertise to confirm that it is of an acceptable scientific standard, however we have significant reservations, as outlined above.

## References

[1] : Global Atlas of palliative care at the end of life.2014;

[2] : Reported availability and gaps of pediatric palliative care in low- and middle-income countries: a systematic review of published data. *J Palliat Med*.2014;17(12) : 10.1089/jpm.2014.0095 1369-83 10.1089/jpm.2014.0095 25225748PMC4268583

[3] : Children's palliative care in low- and middle-income countries. *Arch Dis Child*.2016;101(1) : 10.1136/archdischild-2015-308307 85-90 10.1136/archdischild-2015-308307 26369576

# Peer review: Reviewer response for version 3

**DOI:** 10.21956/wellcomeopenres.17970.r40908

**Published:** 2020-10-26

**Authors:** Chris Feudtner

**Affiliations:** 1 Department of Pediatrics, Children's Hospital of Philadelphia, Philadelphia, PA, USA

In Version 3 of the manuscript, the authors have aligned all key aspects of the paper with the strengths and limitations of their pragmatic assessment of the levels of palliative care services for children in nations around the globe. They present the data they gathered clearly and offer reasonable and thoughtful interpretations, as well as ideas for future research on this topic. As such, I approve of this manuscript’s official indexing.

Is the work clearly and accurately presented and does it cite the current literature?

Yes

If applicable, is the statistical analysis and its interpretation appropriate?

Not applicable

Are all the source data underlying the results available to ensure full reproducibility?

Yes

Is the study design appropriate and is the work technically sound?

Yes

Are the conclusions drawn adequately supported by the results?

Yes

Are sufficient details of methods and analysis provided to allow replication by others?

Yes

Reviewer Expertise:

Pediatric palliative care research, mostly in the context of the United States.

I confirm that I have read this submission and believe that I have an appropriate level of expertise to confirm that it is of an acceptable scientific standard.

# Peer review: Reviewer response for version 2

**DOI:** 10.21956/wellcomeopenres.17713.r39528

**Published:** 2020-08-19

**Authors:** Busisiwe Nkosi

**Affiliations:** 1 Africa Health Research Institute, KwaZulu-Natal, South Africa; 2 Institute for Global Health, University College London, London, UK

The status remains ‘not approved’ based on the methodological limitations raised in the previous report.

Is the work clearly and accurately presented and does it cite the current literature?

Yes

If applicable, is the statistical analysis and its interpretation appropriate?

Not applicable

Are all the source data underlying the results available to ensure full reproducibility?

Yes

Is the study design appropriate and is the work technically sound?

Partly

Are the conclusions drawn adequately supported by the results?

Partly

Are sufficient details of methods and analysis provided to allow replication by others?

Yes

Reviewer Expertise:

Research ethics, ethical and legal challenges in adolescent research; vulnerability in and through research.

I confirm that I have read this submission and believe that I have an appropriate level of expertise to state that I do not consider it to be of an acceptable scientific standard, for reasons outlined above.

# Peer review: Reviewer response for version 2

**DOI:** 10.21956/wellcomeopenres.17713.r39529

**Published:** 2020-08-18

**Authors:** Joanne Wolfe, Rachel Deming

**Affiliations:** 1 Department of Psychosocial Oncology and Palliative Care, Dana-Farber Cancer Institute, Boston, MA, USA; 2 Pediatric Palliative Care, Department of Psychosocial Oncology and Palliative Care, Dana-Farber Cancer Institute, Boston, MA, USA; 3 Pediatric Palliative Care, Boston Children's Hospital, Boston, MA, USA; 4 Pediatrics, Harvard Medical School, Boston, MA, USA

We appreciate the authors’ comprehensive response to our critique. At the same time, we believe that the study’s approach remains scientifically flawed. As noted previously, the study’s validity is greatly impacted by misclassification bias since those invited to respond were not experts in pediatric palliative care. As in all medical specialties, pediatric expertise is independent of adult provider know-how. The authors alo state that it is not possible to rely on pediatric palliative care expertise since such expertise may not even be present in some countries. We would suggest that if no pediatric palliative care expertise exists, that country should be classified as a 1. We agree that knowledge of global development of pediatric palliative care is important to support global advocacy for seriously ill children and their families, and encourage the authors and others to pursue this agenda with scientific rigor.

Is the work clearly and accurately presented and does it cite the current literature?

Yes

If applicable, is the statistical analysis and its interpretation appropriate?

Not applicable

Are all the source data underlying the results available to ensure full reproducibility?

Yes

Is the study design appropriate and is the work technically sound?

No

Are the conclusions drawn adequately supported by the results?

No

Are sufficient details of methods and analysis provided to allow replication by others?

Yes

Reviewer Expertise:

Pediatric Palliative Care.

We confirm that we have read this submission and believe that we have an appropriate level of expertise to confirm that it is of an acceptable scientific standard, however we have significant reservations, as outlined above.

# Peer review: Reviewer response for version 2

**DOI:** 10.21956/wellcomeopenres.17713.r39852

**Published:** 2020-08-18

**Authors:** Chris Feudtner

**Affiliations:** 1 Department of Pediatrics, Children's Hospital of Philadelphia, Philadelphia, PA, USA

The assessment of children’s palliative care around the globe is an important undertaking. Such assessments can inform global and national health policy by identifying opportunities for improvement, and for tracking progress (or regression) over time at moving upward on the developmental scale of implementing high quality pediatric palliative care for children. At the same time, a global assessment including every nation is a daunting task that requires calibration of expectations. As such the success of any such assessment can be gauged as to whether it enables, to a reasonable degree, this ability to spot opportunities and monitor progress.

 In these regards, the project conducted by the authors, and their report, succeed. Their main conclusions, as summed up in the abstract, is that children’s palliative care is not widely available (with high levels availability in less than 10% of the global child-age population), and that the greatest opportunities to improve access are likely to be found in low- and middle-income countries. They also found, in a comparison that they appropriately do not overstate, that some degree of progress appears to have been made over the past decade. Lastly, the authors spot a clear opportunity to continue to improve the methodology by which such global assessments can occur. Even with the clearly stated limitations of the project’s methodology kept in mind, these are reasonable conclusions substantiated by the data and findings from their project.

 I offer 3 suggestions that I think would further strengthen the report.

 First, make clearer from the outset of the report that the data is from key informants in each country, and not objective comparative data. Specifically, change the title to: “Global development of children’s palliative care: An international survey of in-nation expert perceptions in 2017.” This accurately states the data that the study is based on; and as currently written, the manuscript clearly addresses the strengths and limitations of this data. Given this more qualitative than qualitative, more subjective than strictly objective, nature of the data, I am not surprised or overly perturbed that the classification of specific countries may be less than absolutely accurate; the data does not have sufficient precision to offer precise level classifications. The overall conclusions of the project, though, do seem justified by the data.

 Second, reconsider the projection of the Figure 2 map. At the risk of sounding pedantic, the choice of map projection is worthy of though, as each projection emphasizes one attribute while inevitably distorting other attributes. The current map projection is “high latitude biased”, enlarging area nearer to the poles. An equal-area projection (Equal Earth) would be a more appropriate projection, given that one of the most important points of the report is that palliative care for children is not readily available on most of the global landmass. (One might even consider 2 maps, one with equal area, the other with equal population being depicted (see for example here), if the team is able technically to create this alternative projection, as that most important point is about population, not really landmass. This projection would draw attention to the fact that the two most populous countries either do not have palliative care readily available or are “unresolved”, underscoring two opportunities.)

 Third, the authors might be more explicit about what “other sources of evidence” they would want to use to “triangulate” the findings (as they suggest in the second bullet under the “Strengths and Limitations” heading). If more resources were available, what data would they seek to collect? The argument has been made by other reviewers that those experts should, to the degree possible, include children’s palliative care experts; in addition to this, what other steps would the authors take to improve the expert perceptions portion of the evidence? Would they seek to obtain more experts per country? Would they insert a process whereby the in-nation experts would review each other’s assessment and have them converge on a consensus? How would they avoid the “unresolved” problem? And if objective data were to be obtained, what would it be? Would there be different types of data suitable to different levels of palliative care development in the country? In other words, the authors have an opportunity to point the way forward for the next iteration of this work, and more specific suggestions would be potentially quite beneficial in continuing the elevate the grade of evidence from the published literature (as was previously done) to the current survey methodology (with its strengths and limitations) to the next methodological iteration. A paragraph of suggestions would suffice.

Is the work clearly and accurately presented and does it cite the current literature?

Yes

If applicable, is the statistical analysis and its interpretation appropriate?

Not applicable

Are all the source data underlying the results available to ensure full reproducibility?

Yes

Is the study design appropriate and is the work technically sound?

Yes

Are the conclusions drawn adequately supported by the results?

Yes

Are sufficient details of methods and analysis provided to allow replication by others?

Yes

Reviewer Expertise:

Pediatric palliative care research, mostly in the context of the United States.

I confirm that I have read this submission and believe that I have an appropriate level of expertise to confirm that it is of an acceptable scientific standard, however I have significant reservations, as outlined above.

# Response

**Published:** 2020-09-24

**Authors:** David Clelland

**Affiliations:** University of Glasgow, UK

We are extremely grateful to Chris Feudtner for such a sympathetic, insightful and constructive review of Version 2 of our paper. We agree with all three of the points made and propose to implement each of them in version 3 of the paper.

# Peer review: Reviewer response for version 1

**DOI:** 10.21956/wellcomeopenres.17343.r38790

**Published:** 2020-06-17

**Authors:** Busisiwe Nkosi

**Affiliations:** 1 Africa Health Research Institute, KwaZulu-Natal, South Africa; 2 Institute for Global Health, University College London, London, UK

The study addresses an important topic. It focuses on the state of palliative care for children at the global level. The aim of the study was to: “obtain a first global overview of the national development of children’s palliative care based on the knowledge of in-country experts”. It reports on the methodological and conceptual challenges in mapping palliative care for children.

 The paper is well written, and the process of categorisation is well-explained. The study is useful in advocating for children’s palliative care, and to improve indicators to measure it at the country and international levels.

 The methodological and conceptual limitations outweigh the authors’ abilities to map and categorise levels of children’s palliative care development at the national level and globally that they undermine the utility of the study results:

 The study describes the limitations of self-reported data and suggests the triangulation of data sources to address this limitation. However, this could have been considered in this study given that similar work has been done previously.

 On pages 8-9, the authors state: “A significant proportion of countries included in the global mapping survey have been excluded from these results on the basis that participants provided conflicting categorisations that could not be reconciled. The exclusion of these countries limits the extent to which the results presented here can be considered a comprehensive assessment of palliative care development”. The statement is self-defeating and raises questions about the study results.

 The authors state that lack of definition of palliative care for children may have resulted in different interpretations, and this could have resulted in under or over-reporting, making the data unreliable.

 The categorisation of children’s palliative care development is useful, it also illustrates some of the limitations resulting from the reliance on the country experts sampled in the survey. For example, South Africa is categorised as 3a, yet there is an existence of a national palliative care association; and palliative care is provided by multiple providers ranging from community-based, to hospice and hospital-based services. This raises questions about categorisation of palliative care development for some of the countries.

 The authors must be commended for their work in addressing this important yet complex topic. Whilst the study raises important issues on the development of palliative care for children nationally and globally, the methodological and conceptual issues undermine the results.

Is the work clearly and accurately presented and does it cite the current literature?

Yes

If applicable, is the statistical analysis and its interpretation appropriate?

Not applicable

Are all the source data underlying the results available to ensure full reproducibility?

Yes

Is the study design appropriate and is the work technically sound?

Partly

Are the conclusions drawn adequately supported by the results?

Partly

Are sufficient details of methods and analysis provided to allow replication by others?

Yes

Reviewer Expertise:

Research ethics, ethical and legal challenges in adolescent research; vulnerability in and through research.

I confirm that I have read this submission and believe that I have an appropriate level of expertise to state that I do not consider it to be of an acceptable scientific standard, for reasons outlined above.

# Response

**Published:** 2020-06-22

**Authors:** David Clelland

**Affiliations:** University of Glasgow, UK

We thank this reviewer for the attention given to our paper and for acknowledging the importance of our topic, the clarity of our presentation, its value in advocating for children’s palliative care, and its contribution to improving the indicators by which children’s palliative care can be measured.

 The reviewer refers to the ‘methodological and conceptual limitations of the study’ and lists some examples:

 1 The absence of ‘triangulation’ that has been used in previous work. We accept this is a reasonable point, but previous work of this type has been restricted to a much smaller number of countries than those covered here. We did not have the resource to support triangulation on this scale.

 2 What the reviewer sees as a ‘self defeating statement’ about the problems of conflicting responses, we regard as an honest and open statement of limitation which we mitigated by not including the relevant countries in the final analysis.

 The reviewer questions the grading of children’s palliative care in South Africa (3a), making reference to some generic indicators that are not specific to children’s palliative care. It is worth noting that our grading of adult palliative care for South Africa, published elsewhere ( https://www.jpsmjournal.com/action/showPdf?pii=S0885-3924%2819%2930664-5 ) gives a grade of 4a. This is a form of ‘triangulation’ which may indeed demonstrate the veracity of our findings – children’s palliative care in the view of in-country experts in South Africa does not reach a level of development that those same expert source provided across 10 sets of indicators when assessing adult provision.

 Overall, we would suggest that this reviewer’s comments and the assessment that we ‘partly’ addressed two of the key dimensions under review and fully addressed the three other relevant criteria, seems incommensurate with an overall judgement of ‘not accepted’.

# Peer review: Reviewer response for version 1

**DOI:** 10.21956/wellcomeopenres.17343.r38789

**Published:** 2020-06-10

**Authors:** Joanne Wolfe, Rachel Deming

**Affiliations:** 1 Department of Psychosocial Oncology and Palliative Care, Dana-Farber Cancer Institute, Boston, MA, USA; 2 Pediatric Palliative Care, Department of Psychosocial Oncology and Palliative Care, Dana-Farber Cancer Institute, Boston, MA, USA; 3 Pediatric Palliative Care, Boston Children's Hospital, Boston, MA, USA; 4 Pediatrics, Harvard Medical School, Boston, MA, USA

This is a well-written paper on an important and understudied topic with some notable strengths and yet significant limitations. The paper starts strong, clearly explaining the importance of pediatric palliative care and the need for global mapping. The authors appropriately cite prior studies and their limitations, and current gaps in knowledge. At the end of the introduction, they explicitly lay out the objectives of the study and the structure of the rest of the paper, which helps the reader prepare and subsequently follow the information that is to come. 

 Unfortunately, we have some fundamental concerns about the methodology and in turn results of the study. The authors use data from a 2017 questionnaire examining the level of national palliative care development across the globe, which included two questions specific to the development of children’s palliative care. Per the paper, this online survey was completed by “in-country experts actively engaged at the national level in the development, delivery, and co-ordination of palliative care activity.” Importantly, these individuals were not likely experts in pediatric palliative care. Being an expert in adult palliative care does not necessarily mean you have in-depth knowledge of palliative care services available for children. It is problematic that categorization of countries came directly from the responses of these experts. We therefore have serious concerns about the validity of the results. 

 Let us consider pediatric palliative care in the United States (US) as an example. As pediatric palliative care experts based in the US we wonder why it was categorized as 4a rather than 4b given the category descriptors and our knowledge of other countries categorized as such. Specifically, the US has activism for children’s palliative care nationally. For example, as a result of advocacy, in 2010 with the initiation of the Affordable Care Act, policy change led to the opportunity for children to receive disease-directed therapy concurrent with hospice, a provision not available for adults ^1^^,^^2^. The American Board of Pediatrics has recognized Hospice and Palliative Medicine as a formal specialty since 2008. Interdisciplinary pediatric palliative care is now a specialty service available in a majority of freestanding children’s hospitals across the US. Most health professionals, local communities, and members of our society are aware of pediatric palliative care as exemplified by media coverage of these services ^3^^,^^4^. The US has unrestricted access to strong pain-relieving drugs, including morphine. The American Academy of Pediatrics has published guidelines around children’s palliative care ^5^ and multiple academic centers have integrated palliative care into pediatrics residency curricula. Finally, pediatrics is a strong and integral part of the American Academy of Hospice and Palliative Medicine. Despite this level of PPC integration in the US, one of the "in-country experts" ranked the US as a 1 “No known palliative care activity for children” (Extended Data: SurveyResponsesQ26_995). We use the United States to exemplify the potential flaws of relying on adult experts to rank pediatric palliative care development across the globe. While there remains room for further pediatric palliative care integration in the US, this is also the case for the other countries categorized as 4b. And there is substantial misclassification of other countries. For example, Russia was ranked a 4a based on a single survey respondent. Our knowledge of the country is that it should be categorized as a 3a, isolated children’s palliative care provision.

 We do commend the authors for a paper that brings attention to the important issue of mapping children's palliative care across the globe. While we raise serious issues around the methodology and in turn the results, we appreciate the discussion around the complexity and nuances of palliative care in children and the inherent challenges around measuring it globally. As mentioned by the authors, we agree that in order to more accurately capture the current state of pediatric palliative care, there needs to be a global survey using measures and experts specific to children.

Is the work clearly and accurately presented and does it cite the current literature?

Yes

If applicable, is the statistical analysis and its interpretation appropriate?

Not applicable

Are all the source data underlying the results available to ensure full reproducibility?

Yes

Is the study design appropriate and is the work technically sound?

No

Are the conclusions drawn adequately supported by the results?

No

Are sufficient details of methods and analysis provided to allow replication by others?

Yes

Reviewer Expertise:

Pediatric Palliative Care.

We confirm that we have read this submission and believe that we have an appropriate level of expertise to state that we do not consider it to be of an acceptable scientific standard, for reasons outlined above.

## References

[1] : H.R. 3590 (111th): Patient Protection and Affordable Care Act. *(accessed 8 June 2020)*.2009; Reference source

[2] : Health Care Reform and Concurrent Curative Care for Terminally Ill Children: A Policy Analysis. *J Hosp Palliat Nurs*.2011;13(2) : 10.1097/NJH.0b013e318202e308 81-88 10.1097/NJH.0b013e318202e308 22822304PMC3401095

[3] : Lives less ordinary. *The New Yorker*.2014; Reference source

[4] : For kids with cancer, focusing on quality of life. *The New York Times*.2017; Reference source

[5] : Pediatric Palliative Care and Hospice Care Commitments, Guidelines, and Recommendations. *Pediatrics*.2013;132(5) : 10.1542/peds.2013-2731 966-972 10.1542/peds.2013-2731 28448256

# Response

**Published:** 2020-06-22

**Authors:** David Clelland

**Affiliations:** University of Glasgow, UK

We thank the reviewers for their detailed response to our paper and for the ‘notable strengths’ in our work to which they refer. We note that they concur with our own assessment of the limitations of the study and of the need to improve on the methodology we have adopted as we make steps towards a more robust approach to research in a specialised area of palliative care where there remain many challenges.

The reviewers then raise the question of relevant expertise in the respondents to our survey, and take the view that national experts in palliative care will not necessarily have ‘in-depth knowledge of palliative care services available for children’. This is an assertion by the reviewers and cannot constitute a legitimate critique of our method or of our results. We have acknowledged the distinct and perhaps contested status of children's palliative care in our discussion of definitional issues and acknowledged that a survey specifically about children's palliative care would be the desirable next step in developing an understanding of this. But it seems to us unreasonable to discard the views of palliative care experts on the grounds that they are not specialists in children's palliative care, especially when the latter may not even be present in some countries.

The reviewers also question the grading of their own country, the USA, and make reference to the underlying data as an example of the problem they perceive, specifically SurveyResponsesQ26_995. In fact, this response came from the representative of an organisation that works in one of the poorest countries in the world, but who for some reason gave the USA as the host country for the organisation – an issue we noted in some other responses, which we dealt with case by case. This particular questionnaire was therefore quickly excluded from our analysis of the USA data. Moreover, the reviewers may be reassured to know (and we regret that confidentiality principles don’t allow us to give more details) that the other two responses from the USA, not only both gave the same grading (4a), but also came from impeccable organisational sources and were signed off in one case by a senior clinician of global standing in the field of palliative care.

In sum, we reiterate our thanks to the reviewers, who clearly concur with us on so many issues. We contend however that the reviewers make ‘the perfect the enemy of the good’. We know, based on our experience of previous mapping studies we have conducted, that despite the limitations and our gradual efforts to overcome them, these studies have proved enormously valuable to the field. Indeed, they constituted the underlying evidence base for the World Health Assembly Resolution on palliative care, which was passed in 2014, and which of course supports the provision of palliative care across the entire life course. No other group has attempted to map the global development of children’s palliative care in the manner we present in our paper. Until such an attempt is made with an improved methodology, we argue that the current work should be seen, not as definitive, but as the best evidence we currently have, and which moreover is highly likely to be of benefit to the field on which it is focussed. To take a phrase from the paediatrician Winnicott, it is ‘good enough’.
